# Role of the Foliar Endophyte *Colletotrichum* in the Resistance of Invasive *Ageratina adenophora* to Disease and Abiotic Stress

**DOI:** 10.3390/microorganisms12122565

**Published:** 2024-12-12

**Authors:** Ailing Yang, Yuxuan Li, Zhaoying Zeng, Hanbo Zhang

**Affiliations:** 1 State Key Laboratory for Conservation and Utilization of Bio-Resources in Yunnan, Yunnan University, Kunming 650091, China; m18487203674@163.com (A.Y.); 15887818446@163.com (Y.L.); zyzeng_gzu@163.com (Z.Z.); 2 School of Ecology and Environmental Science, Yunnan University, Kunming 650091, China

**Keywords:** plant invasion, plant–fungal interaction, plant pathogen, drought and nutrient stress

## Abstract

Plant-associated fungi often drive plant invasion success by increasing host growth, disease resistance, and tolerance to environmental stress. A high abundance of *Colletotrichum* asymptomatically accumulated in the leaves of *Ageratina adenophora*. In this study, we aimed to clarify whether three genetically distinct endophytic *Colletotrichum* isolates (AX39, AX115, and AX198) activate invasive plant defenses against disease and environmental stress. We observed that, in the absence of pathogen attack and environmental stress, the foliar endophyte *Colletotrichum* reduced photosynthesis-related physiological indicators (i.e., chlorophyll content and soluble sugar content), increased resistance-related indicators (i.e., total phenolic (TP) and peroxidase (POD) activity), and decreased the biomass of *A. adenophora*. However, endophytic *Colletotrichum* strains exhibit positive effects on resistance to certain foliar pathogen attacks. Strains AX39 and AX115 promoted but AX198 attenuated the pathogenic effects of pathogen strains G56 and Y122 (members of *Mesophoma ageratinae*). In contrast, AX39 and AX115 weakened, but AX198 had no effect on, the pathogenic effect of the pathogen strain S188 (*Mesophoma speciosa*; Didymellaceae family). We also found that endophytes increase the biomass of *A. adenophora* under drought or nutrient stress. Strain AX198 significantly increased stem length and chlorophyll content under drought stress. Strain AX198 significantly increased the aboveground dry weight, AX115 increased the stem length, and AX39 significantly increased the chlorophyll content under nutrient stress. Our results revealed that there are certain positive effects of foliar *Colletotrichum* endophytes on *A. adenophora* in response to biotic and abiotic stresses, which may be beneficial for its invasion.

## 1. Introduction

Endophytes are a group of microorganisms (bacteria or fungi) that reside in the internal tissues of plants in symbiotic associations without causing any disease symptoms [1,2,3]. Among endophytes, endophytic fungi are among the important components of plant–microorganism interaction systems. Endophytic fungi include Chytridiomycota, Zygomycota, Ascomycota, and Basidiomycota, which occur in diverse environments [4]. On the basis of their lifestyle, functional diversity, biology, and mode of transmission, endophytic fungi are classified as systemic/true endophytes (mutualistic for the entirety of their lifespan) or transient/nonsystemic endophytes (adapting transient modes of lifestyles, mutualistic and parasitic being the two most common modes, during different life cycle stages) [5]. The mutualistic relationships of these endophytic fungi with host plants have profound beneficial impacts on plant communities, such as improving plant fitness, increasing tolerance to biotic and abiotic stresses, and increasing plant biomass. In turn, the host plant provides fungal endophytes with refuge and nutrients and allows them to survive within the next generation of host plants.

The association of a plant with endophytic fungi is, therefore, a supreme beneficial relationship for both partners, especially the plant. Fungal endophytes can stimulate the growth of plants directly or indirectly by triggering the solubilization of phosphate, potassium, and ions [6,7]. Endophytic fungi also confer resistance against plant pathogens through enhanced production of plant secondary metabolites, endophyte-specific metabolite synthesis, induction of systemic or local immune responses, or competitive elimination of pathogens [8]. During their life cycle, plants are subjected to various abiotic stresses, such as drought stress, temperature stress, salinity stress, and heavy metal toxicity stress [9]. In addition to their intrinsic mechanism of adaptation, endophytes enhance the alleviation of the negative effects of stress conditions faced by host plants through two mechanisms: (a) activation of the stress response after host exposure and (b) the synthesis of antistress biochemicals by endophytes. The antistress biochemicals, which function as ROS scavengers, include the enzymes superoxide dismutase (SOD), catalase (CAT), and ascorbate- or thiol-dependent peroxidase (APX). For example, Piriformospora indica induces salt tolerance in barley by increasing the levels of antioxidants [10].

Endophytic fungi have similar important ecological functions in invasive ecosystems, and the presence or absence of mutualist fungi may partially explain why some alien plants become invasive [11]. First, endophytic fungi can directly act on invasive plants to improve their growth, reproduction, defense against pathogens and herbivores, and environmental tolerance. For example, Aschehoug et al. [12] reported that inoculation with the endophyte *Alternaria* sp. strain CID120 could directly promote the growth of the invasive plant *Centaurea stoebe*. Endophytic fungi can improve the reproductive success of invasive plants by increasing germination [13] or facilitating the allocation of plant resources to adopt more efficient reproductive strategies [14]. For example, endophytic infection of *Cyperus rotundus* plants reduces investment in expensive sexual reproductive structures (inflorescence) but increases investment in the primary mode of reproduction (vegetative tuber production). This trade-off may increase population growth and thus establish success [15]. The leaves of the invasive annual *Anthemis cotula* have endophytic microorganisms with plant growth-promoting traits, such as ammonia production, indole acetic acid (IAA) production, phosphate dissolution, and biocontrol of active substances [16]. Currie et al. reported that foliar endophytic fungi (*Colletotrichum acutatum*, *Alternaria alternata*, and *Cladosporium oxysporum*) can effectively reduce the pathogenicity of *Puccinia komarovii*, a rust pathogen, to the invasive plant *Impatiens glandulifera* [17]. The invasive grass *Cirsium arvense* was inoculated with the endophyte *Chaetomium cochliodes* to produce oxolipin and jasmonate metabolites, which induced the production of plant defense metabolites for systemic resistance [18]. Rudgers and Clay reported that *Neotyphodium coenophialum* significantly reduced the abundance and diversity of herbivorous insects in the invasive *Lolium arundinaceum* [19]. The survival rate of *Phragmites australis* seedlings under salt stress could be improved by inoculation with endophytes from the dark septum (DSE). When exposed to salt stress, *P. australis* can establish reciprocal relationships with DSEs [20].

In addition, endophytic fungi can indirectly increase the competitive advantage of invasive plants by interfering with native plants; for example, they can change the soil nutrient content and soil through the cycle of endophytic fungal species to inhibit the growth of native plants and indirectly promote the development of their own species [21]. Arbuscular mycorrhizal fungi (AMF) can also reduce the dependence of host plants on symbiosis with AMF without changing their biomass and indirectly promote the establishment of the host grass and subsequent invasion [22].

*Ageratina adenophora* (Sprengel) R.M. King & H. Robinson is a perennial herb of Asteraceae native to the Mexican area of America. This plant has invaded more than 40 tropical to temperate regions worldwide, causing severe ecological impacts and economic losses [20]. Since the invasion of Yunnan Province from the China–Myanmar border in the 1940s, *A. adenophora* has been widely distributed in Yunnan Province and neighboring Sichuan, Guizhou, Guangxi, Xizang, and other provinces [23], and is one of the 18 most severely invasive plants in China [24]. *A. adenophora* can associate with beneficial microorganisms (such as nitrogen-fixing bacteria) around its roots to gain a sustained growth advantage [25,26]. Moreover, some bacteria have a more significant growth-promoting effect on *A. adenophora* than on local plants [27]. *A. adenophora* can also accumulate large amounts of native plant pathogenic fungi in its leaf tissue in the form of endophytic fungi, which may facilitate successful invasion [28]; these dominant genera of foliar endophytic fungi include *Colletotrichum* sp., *Nemania* sp., *Phomopsis* sp., and *Xylaria* sp. [29].

Previously, some *Colletotrichum* spp. endophytes were shown to have a weak negative impact on the growth of *A. adenophora* planted in agricultural soil and forest soil but can increase the pathogenicity of the foliar pathogen *Diaporthe helianthi* and slightly reduce the herbivory of *A. adenophora* seedlings in the wild [30]. Species of *Colletotrichum* sp. are commonly found in many plant hosts, such as pathogens, endophytes, and, occasionally, saprobes. It has been reported that *Colletotrichum*, an endophyte, is present in *Dendrobium orchids* [31], *Taxus mairei* [32], *Citrus grandis* cv. “Tomentosa” [33], tea plants [34], and wild banana plants (*Musa acuminata*) [35]. *Colletotrichum* plays both pathogenic and endophytic roles depending on the genetics of the fungus, the interaction between *Colletotrichum* and the specific host, and biotic and abiotic factors in the environment [36]. *A. adenophora* can be infected frequently by diverse foliar pathogens in its invaded range, particularly members of the family Didymellaceae [37]. Didymellaceae species are distributed throughout a broad range of environments, and most are economically important plant fungal pathogens with a wide host range [38]. Our previous study characterized the virulence and host range of strains in the family Didymellaceae, which includes major genera (*Didymella*, *Epicoccum*, *Remotididymella*, and *Mesophoma*). The results show that strains of the genus *Mesophoma* are *A. adenophora* host-specific pathogens with weak virulence to native plants but strong virulence to the host *A. adenophora*, highlighting their potential as biocontrol agents for *A. adenophora* invasion [37]. It is unclear whether the widely occurring foliar *Colletotrichum* endophytes also increase the pathogenicity of the dominant foliar pathogens from the family Didymellaceae. Moreover, although some *Colletotrichum* endophytes have a weak negative impact on the growth of *A. adenophora* [30], it is necessary to determine whether they can increase host plant resistance to environmental stressors such as drought stress or nutrient stress because *A. adenophora* occurs in the wild in harsh environments (roadsides and railway embankments), which are characterized by drought and low nutrient availability [21].

In this study, we aimed to compare the role of three genetically distinctive *Colletotrichum* strains in the resistance of invasive *A. adenophora* to the pathogenicity of three foliar pathogen members from the Didymellaceae family, as well as to abiotic stress, including drought and nutrient stress. We hypothesized that, compared with plants inoculated with *A. adenophora* without endophytes, those inoculated with *Colletotrichum* would (1) have greater biomass, (2) decrease the pathogenicity of the pathogen on their leaves, and (3) alleviate the effects of drought stress and nutrient stress on plant growth.

## 2. Materials and Methods

### 2.1. Description of Fungi

The three *Colletotrichum* strains, AX39, AX198, and AX115, used in this study were previously isolated from healthy leaves of *A. adenophora* in Yunnan Province, China. The ITS sequences of the strains were searched for similar DNA sequences in GenBank via BLAST, and the results revealed that both AX39 (GenBank accession number: PP227195) and AX198 (GenBank accession number: PP227202) presented the highest ITS sequence similarity with the *C. gloeosporioides* complex, whereas AX115 (GenBank accession number: KC507204) presented the highest ITS sequence similarity with the *C. acutatum* complex [39,40]. The three Didymellaceae strains, G56, Y122, and S188, used in this study were previously collected from diseased leaves, healthy leaves, and roots of *A. adenophora*, respectively [37]. They are leaf pathogens of *A. adenophora* and have strong virulence to the host according to the pathogenicity assay [37]. Among them, G56 and Y122 are associated with *Mesophoma ageratinae*, and S188 is associated with *Mesophoma speciosa* [41].

### 2.2. Growth Effects of Colletotrichum on Its Host A. adenophora

#### 2.2.1. Planting the Host *A. adenophora* and Inoculation with *Colletotrichum*

The effects of three *Colletotrichum* strains (AX39, AX115 and AX198) on the growth of *A. adenophora* were first tested. In addition to the control treatment without inoculation of *Colletotrichum* strains, there were 4 groups in the experiment, and 5 potted plants were planted in each group as replicates, for a total of 20 potted plants. Seeds of *A. adenophora* collected from wild plants in Xishan Forest Park, Kunming, were immersed in 75% ethanol for 10 min and then in 3% sodium hypochlorite for 10 min and then washed three times with sterile water. The seed surface disinfection method was modified on the basis of the method described by Arnold and Lutzoni [42], which ensures that the disinfection was thorough (the last clean sterile water was applied on PDA medium, and no colony growth was observed after culture at 28 °C, indicating thorough disinfection of the surface), and did not affect the seed germination rate and did not damage the seed embryo (no difference from the germination rate of undisinfected seeds). The surface-disinfected seeds were soaked in sterile water for 12 h, and 4 seeds were cultured in Murashige and Skoog (MS) medium tissue culture bottles with tweezers in an artificial climate chamber (RXZ-380D, Ningbo Southeast Instrument Co., Ltd., Ningbo, China) for one month to germinate seedlings (25 °C ± 1, relative humidity 80% ± 5, photoperiod 12 h, light intensity 12,000 lux) (Figure S1a). These cultivation conditions are related to natural environments where *A. adenophora* and its endophytes typically grow.

For preparation of the spore suspension, the purified fungus was inoculated into 50 mL of sterilized PDB liquid media (200 g potato, 20 g glucose, 1000 mL water, and pH = 5.6 ± 0.2) in 200 mL conical flasks and cultured at 28 °C with shaking for 3–5 days (Figure S1b). After many spores were produced in the PDB culture medium, the mycelia in the PDB culture medium were filtered and removed with 4 layers of sterilized lens paper [43], and the spores were collected by centrifugation of the filtered spore mixture. A spore suspension was prepared by adding 0.5% sterilized gelatin solution, and the concentration was adjusted to ~10^6^ CFU/mL for each strain. The spore suspension was stored at 4 °C until use.

For inoculation, the seedling leaves were sprayed on both sides with a 0.5% gelatin suspension with 5 × 10^6^ CFU/mL spores of *Colletotrichum* strains (1 mL per bottle). The plants in the control group were sprayed with 0.5% sterilized gelatin solution. After 24 h of culture in the chamber, the same inoculation procedure was performed to ensure adequate inoculation. Fungal infection of leaves was detected via the method [42] within three days after inoculation. Three leaves were picked from each plant (at the bottom, middle, and top of the plant), and the middle part of the leaves (avoiding the vein part) was cut into 2 mm^2^ pieces. The surface was disinfected (soaked in 0.5% sodium hypochlorite solution for 2 min, soaked in 75% ethanol for 2 min, washed with sterile water three times, and dried with sterilized filter paper). Nine small pieces (2 mm^2^) were placed on 2% MEA media (0.6 g of malt extract, 0.06 g of soybean peptone, 20 g of agar powder, and 1000 mL of water) at room temperature. Three plates of medium were used for each treatment. After 3–5 days, the number of small leaves with fungal growth was recorded, the fungi were cultured, and DNA was extracted to verify that they were originally inoculated strains.

The inoculated plants were transplanted to polypropylene (PP) cups filled with 100 g of sterilized Danish humus soil (Pindstrup sphagnum, Pindstrup Mosebrug A/S, Kongerslev, Denmark) and 100 mL of water and placed in a plant growth chamber to grow for 1 month (Figure S1c). Afterward, the plants were watered with 50 mL of sterile water every two days and 50 mL of Hoagland nutrient solution once a week. The PP cups were sealed with a PTFE membrane to block air microorganisms. The plants were subsequently transplanted to a pot with an outer diameter of 23.5 cm and a height of 14 cm and cultured in a greenhouse for 2 months (Figure S3). A volume of 200 mL of sterile water was used to irrigate the plants every two days, and 200 mL of Hoagland nutrient solution was used to irrigate the plants once a week. Fungal infection of leaves was tested again in the 4th and 9th weeks during this period.

#### 2.2.2. Measuring Physiological Traits and Growth Indices

The following plant physiological traits were measured via colorimetry kits: chlorophyll content (chlorophyll tester, Shandong Fangke Instrument Co., Ltd., Weifang, China, model FK-YL01), total sugar content (Model A145-1-1 plant soluble sugar content test kit (colorimetric method), Nanjing Jiancheng Bioengineering Institute, Nanjing, China), peroxidase (POD) activity (A084-3-1 peroxidase (POD) assay kit (colorimetric method)), and total phenol (TP) content (A143-1-1 plant total phenol test box (colorimetric method)). The mature leaves of each pot of plants were selected, and 0.2 g of leaves from each sample were weighed, washed with distilled water, dried with filter paper, thoroughly ground after adding liquid nitrogen, and processed according to the methods of different kits. The data of the corresponding wavelengths were subsequently measured with a spectrophotometer to calculate the content of each index.

We measured the unit leaf area dry weight for the 2nd and 5th pairs of leaves from the top to the bottom of *A. adenophora*. We punched five leaf discs with an area of approximately 0.5 cm^2^ for each leaf via an 8 mm diameter stopper borer. These leaf discs were dried for 48 h at 65 °C in an oven to obtain dry weights, after which the unit leaf area of dry matter weight was calculated. All the plants were removed from the soil to clean the roots, and the attached water was blotted dry with filter paper. The stems and roots were cut, and the stem length, root length, and branch number of the plants were recorded. After drying at 60 °C for 48 h, the aboveground dry weight, underground dry weight, and root–to-shoot ratio of the plants were obtained by combining the leaf dry weights harvested earlier.

### 2.3. Resistance to Pathogens on A. adenophora Leaves Caused by the Endophyte Colletotrichum

In the above experiment described in Section 2.2, *A. adenophora* plants were inoculated with the endophytic fungi AX39, AX115, and AX198, whereas the control plants did not receive any endophytic fungi. After the physiological indices were measured in the experiment, one week before the plant biomass was harvested, we selected 4 leaves from the fourth pair of equal areas for each pot of plants and inoculated them with the pathogenic fungi S188, G56, and Y122 from the Didymellaceae family and the control group (agar without fungal mycelia). These fungi can successfully cause leafspot symptoms by wounding inoculation when *A. adenophora* seedlings are cultured for 7 days in an artificial climate chamber (25 °C ± 1, relative humidity 80% ± 5, photoperiod 12 h, light intensity 12,000 lux), a condition similar to natural environments where *A. adenophora* and its endophytes typically grow (see description above).

Inoculation with the pathogen was performed as described previously [44]. Briefly, the pathogens were cultured on PDA media for 7 days, after which agar discs (6 mm diameter) containing fungal mycelia were inoculated into the fourth pair of leaves in each pot. There were 5 potted plants in each group. Small wounds were made by lightly touching the underside of the leaf with a sterilized toothpick, resulting in a wound area of ~0.2 cm^2^. The inoculum agar was pressed against the wound on the underside of the leaf using Scotch tape and clipped in place with a bent hair clip. Control inoculation was performed with agar without fungal mycelia. After allowing for a period of 7 days for disease development, observations were made on the leaf surfaces, and the area of the diseased spot was measured. The size of the diseased spot was used as an indicator to evaluate the effect of endophytic fungal inoculation on the resistance of *A. adenophora* to pathogen infection [44].

### 2.4. Resistance of A. adenophora to Drought Stress and Nutrient Stress by the Endophyte Colletotrichum

Sterile and endophyte-infected *A. adenophora* seedlings were planted and prepared as described in Section 2.2. These plants were divided into four groups: the control treatment without endophytes and the inoculation treatment with the AX39, AX115, and AX198 strains. At the same time, the soil was divided into three groups: normal treatment, drought stress treatment [the soil was watered with 100 mL/cup 20% PEG-6000 solution for the first time (drought stress, 20), with an osmotic potential of −4.906 bars to produce drought stress] [45], and nutrient stress treatment (the whole process without supplying nutrient solution), with five pots in each treatment as replicates, for a total of 60 pots of plants. After the plants were irrigated with sterile water according to the water requirements, Hoagland nutrient solution was added once a week for the normal and drought treatments, while only sterile water was added for the nutrient stress treatments. After 45 days of growth in an artificial climate chamber, the plants were harvested, and the chlorophyll content, branch number, root length, stem length, aboveground and underground dry weight, and root shoot ratio of the plants were measured as described in Section 2.2 above.

### 2.5. Data Analysis

The fungal infection status of the leaves was expressed as the isolation rate (number of small leaves with fungal growth per plate/the total number of small leaves per plate × 100%). The response index (RI) was calculated to evaluate the direction (i.e., positive or negative) and intensity of the fungal effect by calculating physiological indices, biomass data, and leaf spot area. The formula is as follows: (treatment-control)/control [46]. The Kolmogorov–Smirnov test was used to determine the normality of the data, and the nonparametric Mann–Whitney U test (for two groups) and Kruskal–Wallis test (for three or more groups) were used to test between-group differences for the data that were not normally distributed. For normally distributed data, analysis of variance (ANOVA) and post hoc comparisons were used to evaluate the differences between groups. Independent sample T tests (2 groups) and Duncan tests and Dunnett T3 tests were used for post hoc multiple comparisons (three or more groups) to test for differences between groups. Normality tests, homogeneity of variance tests, one-way analysis of variance, post hoc multiple comparisons, independent sample T tests, and nonparametric analyses were performed via SPSS v.25.0 (IBM, Chicago, IL, USA). The data were plotted in GraphPad Prism 7 (GraphPad Software, Inc., La Jolla, CA, USA).

## 3. Results

### 3.1. Effects of Colletotrichum on Host A. adenophora Growth

To determine whether these endophytes can successfully colonize the leaves of the host, we first detected the isolation rate of the leaves of *A. adenophora* at 1, 4, and 9 weeks after *Colletotrichum* inoculation (Figure S2). The isolation rates of AX39, AX115, and AX198 decreased with increasing host growth time, with average isolation rates of 69.45%, 66.67%, and 76.85%, respectively, in the first week and decreasing to 14.58%, 36.67%, and 14.17%, respectively, in the ninth week. The isolation rates of strains AX39 and AX198 decreased significantly in the ninth week compared with those in the first week, which may be because the strains transferred to other tissues of the plant along with plant metabolism or reached a balance between strains and plants in the later period. There was no significant difference in colonization among AX39, AX198, and AX115 in each period.

**Figure 1:**
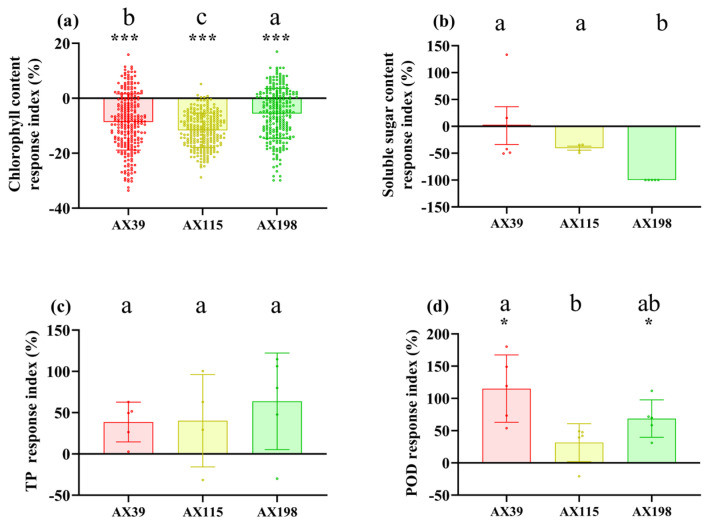
Physiological indices of *A. adenophora* inoculated with endophytic *Colletotrichum* strains. (**a**) Chlorophyll content, (**b**) soluble sugar content, (**c**) total phenol content, and (**d**) peroxidase activity. Dots with different colors represent the raw data of each sample inoculated with the *Colletotrichum* AX39, AX115, and AX198 strains. The RI represents the response index, where the negative RI in panels (**a**,**b**) indicates reduced chlorophyll content and soluble sugar content in the treatment with *Colletotrichum* spp. infection compared with the control without *Colletotrichum* spp. infection. The positive RIs in panels (**c**,**d**) indicate increased total phenol content and peroxidase POD activity in the treatment with *Colletotrichum* spp. infection compared with the control without *Colletotrichum* spp. infection. Nonparametric Mann–Whitney U tests or independent sample T tests were used to identify the differences between each treatment group and the control group (* <0.05, *** <0.001). Post hoc comparisons were performed via Duncan’s test for equal variance and Dunnett’s test for unequal variance (T3 test) to determine whether the difference in the RI was significant among the treatments inoculated with AX39, AX115, and AX198, with different letters indicating significant differences. The error bars are the standard errors.

**Figure 2:**
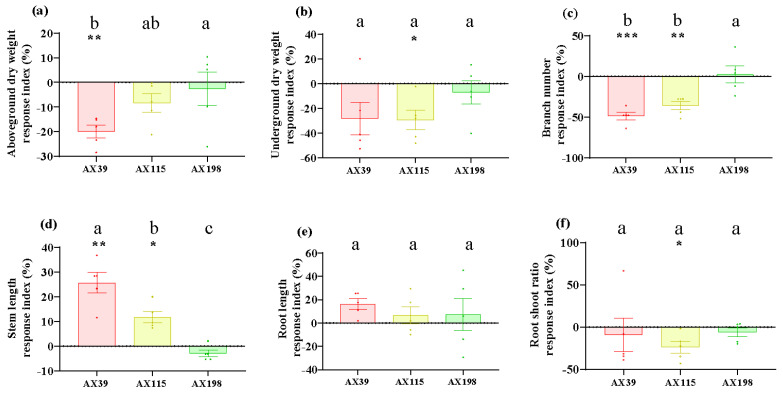
Biomass of *A. adenophora* inoculated with endophytic *Colletotrichum* strains. (**a**) Aboveground parts, (**b**) underground parts, (**c**) branch number, (**d**) stem length, (**e**) root length, and (**f**) root–to-shoot ratio. Dots with different colors represent the raw data of each sample inoculated with the *Colletotrichum* AX39, AX115, and AX198 strains. A negative RI indicates a reduced biomass of *A. adenophora* in the experimental treatment with *Colletotrichum* spp. infection compared with that in the control without *Colletotrichum* spp. infection. Nonparametric Mann–Whitney U tests or independent sample T tests were used to identify the differences between each treatment group and the control group (* <0.05, ** <0.01, *** <0.001). Post hoc comparisons were performed via Duncan’s test for equal variance and Dunnett’s test for unequal variance (T3 test) to determine whether the difference in the RI was significant among the treatments inoculated with AX39, AX115, and AX198, with different letters indicating significant differences. The error bars are the standard errors.

**Figure 3:**
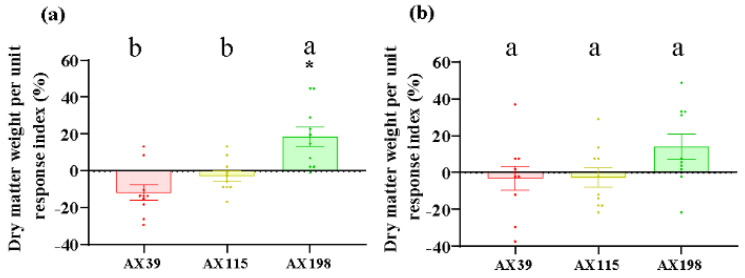
LMA (dry weight per unit area) of *A. adenophora* inoculated with endophytic *Colletotrichum* strains. (**a**) The second pair of leaves, (**b**) the fifth pair of leaves. Dots with different colors represent the raw data of each sample inoculated with the *Colletotrichum* AX39, AX115, and AX198 strains. A negative RI indicates a reduced LMA of *A. adenophora* in the experimental treatment with *Colletotrichum* spp. infection compared with that in the control without *Colletotrichum* spp. infection. Nonparametric Mann–Whitney U tests or independent sample T tests were used to identify the differences between each treatment group and the control group (* <0.05). Post hoc comparisons were performed via Duncan’s test for equal variance and Dunnett’s test for unequal variance (T3 test) to determine whether the difference in the RI was significant among the different treatments inoculated with AX39, AX115, and AX198, with different letters indicating significant differences. The error bars are the standard errors.

Compared with those without inoculation, inoculation with AX39, AX115, and AX198 reduced plant photosynthesis-related physiological indices (i.e., chlorophyll content and soluble sugar content) (Figure 1a,b) and increased plant resistance-related physiological indices [i.e., total phenolic (TP) and peroxidase (POD) activity] (Figure 1c,d). The chlorophyll contents of the plants inoculated with the three strains were significantly (*p* < 0.001) lower than those of the non-inoculated plants, and the POD activities of the plants inoculated with AX39 and AX198 were significantly greater than those of the noninoculated plants. AX39 and AX115 had greater effects on reducing the chlorophyll content and total sugar content than AX198 did. Inoculation with fungal endophytes also reduced plant biomass (aboveground dry weight, underground dry weight, number of branches, and the root–to-shoot ratio) but increased stem length and root length (Figure 2a–f). Among them, both AX39 and AX115 commonly had greater response indices for aboveground biomass and stem length than AX198 did; moreover, AX39 and AX115 adversely affected the LMA (dry matter weight per unit leaf area) (g·m^−2^) of the second pair and the fifth pair of leaves, but AX198 positively affected the LMA of *A. adenophora* (Figure 3a,b).

### 3.2. Resistance of A. adenophora to Foliar Pathogens via Inoculation with the Endophyte Colletotrichum

**Figure 4:**
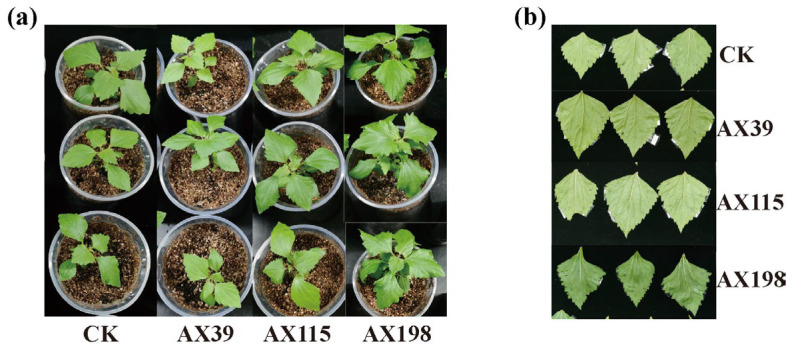
The asymptomatic leaves of *A. adenophora* plants inoculated with a *Colletotrichum* spore mixture (**a**) and wounded and inoculated with agar discs of *Colletotrichum* (**b**). “CK” represents the control group without *Colletotrichum* inoculation.

**Figure 5:**
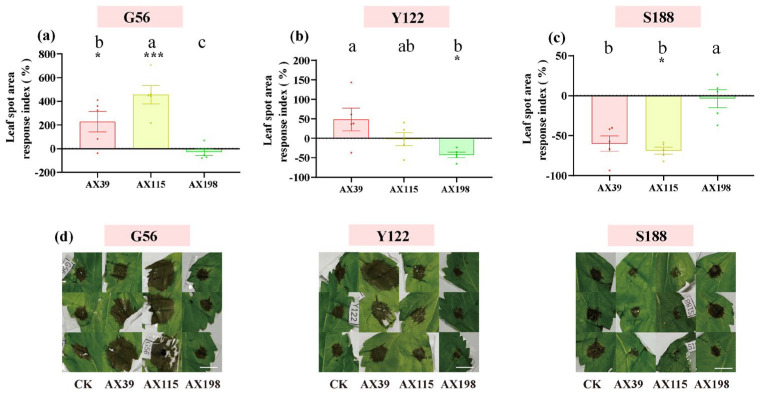
Pathogenicity effects of inoculating endophyte *Colletotrichum* strains on *A. adenophora* after challenge with the (**a**) pathogen G56, (**b**) pathogen Y122, and (**c**) pathogen S188. Dots with different colors represent the raw data of each sample inoculated with *the Colletotrichum* AX39, AX115, and AX198 strains. The specific leaf spot area and morphology are shown in (**d**); scale bar = 10 mm, and “CK” represents the control group without *Colletotrichum* inoculation. Nonparametric Mann–Whitney U tests or independent sample T tests were used to identify the differences between each treatment group and the control group (* <0.05, *** <0.001). Post hoc comparisons were performed via Duncan’s test for equal variance and Dunnett’s test for unequal variance (T3 test) to determine whether the difference in the RI was significant among the different treatments inoculated with AX39, AX115, and AX198, with different letters indicating significant differences. The error bars are the standard errors.

The host *A. adenophora* showed no leaf spot when inoculated with any of the three *Colletotrichum* strains by either spraying the spore suspension (Figure 4a) or wounding and inoculating the plants with agar discs of *Colletotrichum* (Figure 4b). We subsequently tested the resistance of *A. adenophora* inoculated with the endophyte *Colletotrichum* to three foliar pathogens from the *Didymellaceae* family (Figure 5). With respect to infection by the foliar pathogens G56 and Y122 (members of *Mesophoma ageratinae* (Didymellaceae family)), inoculation with the AX39 and AX115 endophytes resulted in a larger leaf disease spot area than inoculation without endophytes, suggesting that inoculation with AX39 and AX115 promoted infection by G56 and Y122 (Figure 5a,b,d). In contrast, the leaf disease spot area in the group inoculated with the AX198 endophyte was smaller than that in the uninoculated group, indicating that AX198 attenuated the pathogenic effects of the G56 and Y122 pathogens (Figure 5a,b,d). With respect to infection by the pathogen *Mesophoma speciosa* (Didymellaceae family) S188, the areas of leaf disease spots inoculated with AX39 and AX115 were smaller than those inoculated with the uninoculated endophyte, indicating that AX39 and AX115 weakened the pathogenic effect of the pathogen S188; however, AX198 had no effect on S188 infection (Figure 5c,d).

### 3.3. Resistance of A. adenophora Inoculated with the Endophyte Colletotrichum to Drought Stress and Nutrient Stress

**Figure 6:**
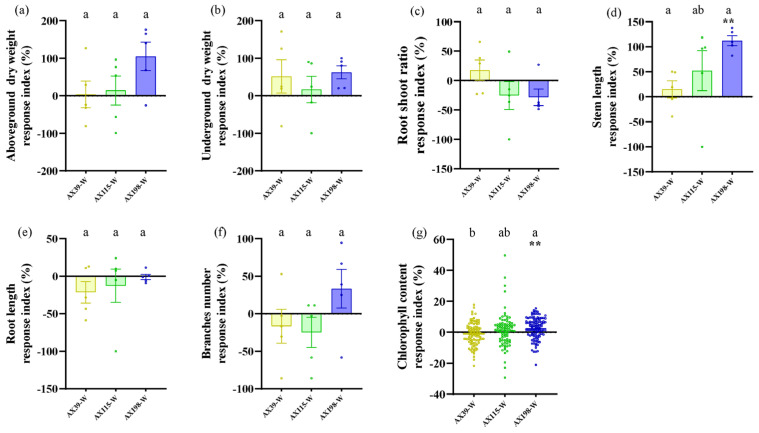
Biomass and chlorophyll content of *A. adenophora* inoculated with endophyte *Colletotrichum* strains under normal conditions and drought stress. (**a**) Aboveground parts, (**b**) underground parts, (**c**) root/shoot ratio, (**d**) stem length, (**e**) root length, (**f**) branch number, and (**g**) chlorophyll content. A positive RI indicates an increased biomass of *A. adenophora* in the drought stress (−W) treatment with *Colletotrichum* strain (AX39, AX115, or AX198) inoculation compared with that without *Colletotrichum* inoculation. The formula is as follows: (treatment_Wcontrol_W)/control_W. Nonparametric Mann–Whitney U tests or independent sample T tests were used to identify the differences between each drought stress treatment group and the normal treatment group (** <0.01). Post hoc comparisons were performed via Duncan’s test for equal variance and Dunnett’s test for unequal variance (T3 test) to show that the differences in the RIs were significant among the treatments inoculated with strains AX39, AX115, and AX198, with different letters indicating significant differences. The error bars are the standard errors.

**Figure 7:**
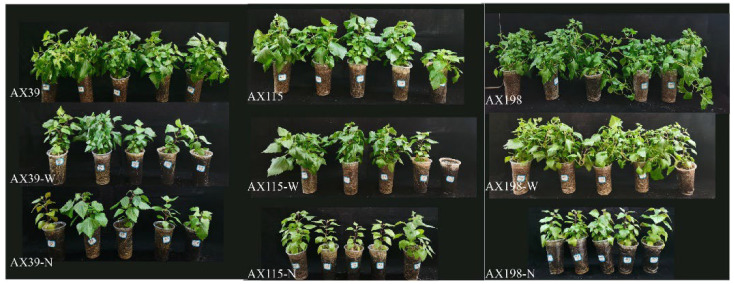
Growth effects of *A. adenophora* inoculated with endophyte *Colletotrichum* strains under nutrient stress and drought stress. Individuals of *A. adenophora* were inoculated with AX39, AX115, or AX198 and grown for one month in a plant growth chamber under nutrient stress (−N) and drought (−W).

**Figure 8:**
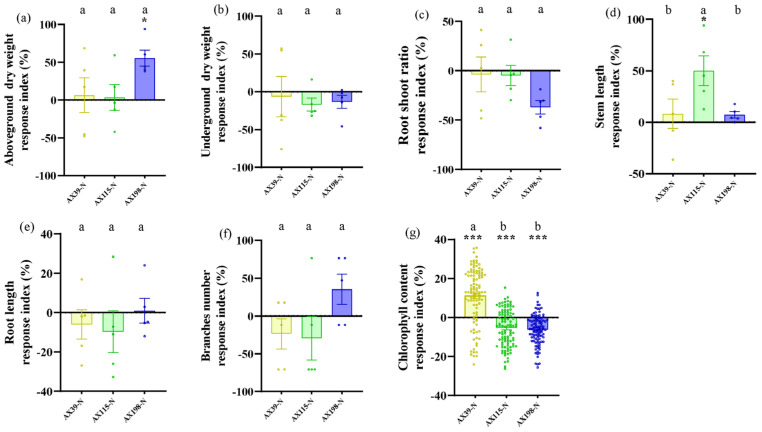
Biomass and chlorophyll content of *A. adenophora* plants inoculated with endophytic *Colletotrichum* strains under normal conditions and nutrient stress conditions. (**a**) Aboveground parts, (**b**) underground parts, (**c**) root/shoot ratio, (**d**) stem length, (**e**) root length, (**f**) branch number, and (**g**) chlorophyll content. A positive RI indicates an increased biomass of *A. adenophora* in the nutrient stress (−N) treatment with *Colletotrichum* strain (AX39, AX115, or AX198) inoculation compared with that without *Colletotrichum* inoculation. The formula is as follows: (treatment_Ncontrol_N)/control_N. Nonparametric Mann–Whitney U tests or independent sample T tests were used to identify the differences between each nutrient stress treatment and the normal treatment (* <0.05,*** <0.001). Post hoc comparisons were performed via Duncan’s test for equal variance and Dunnett’s test for unequal variance (T3 test) to show that the differences in the RIs were significant among the treatments inoculated with strains AX39, AX115, and AX198, with different letters indicating significant differences. The error bars are the standard errors.

Compared with *A. adenophora* without endophyte inoculation, inoculation with the AX198 strain significantly increased stem length (Figure 6d and Figure 7) and chlorophyll content (Figure 6g and Figure 7) under drought stress; inoculation with AX198 significantly increased aboveground dry weight (Figure 7 and Figure 8a), and inoculation with AX115 increased stem length (Figure 7 and Figure 8d) under nutrient stress. In contrast to those under drought stress, inoculation with AX39 significantly increased the chlorophyll content, whereas inoculation with both AX115 and AX198 drastically decreased the chlorophyll content under nutrient stress (Figure 7 and Figure 8g).

## 4. Discussion

Understanding the function of fungal endophytes associated with invasive plants has become an increasing concern of invasion biologists. In general, recently introduced fungal endophytes can directly help invasive plants improve growth, reproduction, defense against pathogens and herbivores, and environmental tolerance [12,13,14,17]. Previously, a high abundance of the endophytic fungus *Colletotrichum* was found to be enriched in the leaves of *A. adenophora* [29]. In this study, we further tested the effects of these endophytic fungi on the growth of *A. adenophora* under pathogen challenge and environmental stress conditions. (1) The host *A. adenophora* did not grow well after asymptomatic infection with three endophytic *Colletotrichum* strains, as indicated by reduced plant photosynthesis-related physiological indices (chlorophyll content and soluble sugar content), increased plant resistance-related physiological indices [total phenolic (TP) and peroxidase (POD) activity], and decreased biomass. (2) Endophytic *Colletotrichum* fungi had some positive effects on the resistance of the leaves of *A. adenophora* to pathogens, but genetically distinct endophytic fungi presented different responses to different pathogens. (3) Under drought or nutrient stress, the endophyte *Colletotrichum* promoted the growth of *A. adenophora*, especially in terms of increasing stem length and aboveground biomass and regulating the leaf chlorophyll content.

In contrast to Expectation 1, although the leaf of the host *A. adenophora* was not affected by inoculation with any of the strains of *Colletotrichum* inoculated via spore suspension (Figure 4a) or by wounding and inoculation via agar discs (Figure 4b), inoculation with *Colletotrichum* had adverse effects on the photosynthetic efficiency and growth of *A. adenophora* (Figure 1a,b and Figure 2). Photosynthesis is considered one of the main driving forces of plant growth, and leaf pathogens affect one of the most important physiological processes. Many studies have shown that pathogen infection results in a decrease in the photosynthetic rate and photosynthetic unit changes, and these changes may be due to cutting or damage to the photosynthetic apparatus [47]. Photosynthesis decreases in bean plants infected with *Colletotrichum lindemuthianum* [48], and photosynthesis decreases in acai leaves infected with *Colletotrichum* [47]. Moreover, it was demonstrated that *Monographella albescens* was able to impair the photosynthetic process in both symptomatic and asymptomatic leaf areas [49]. In fact, many pathogens may impair photosynthesis in asymptomatic, although colonized, tissues, i.e., often called virtual lesions [50,51,52]. Therefore, inoculation with the endophytic fungus *Colletotrichum* inhibited photosynthesis in *A. adenophora* to some extent, leading to a further reduction in biomass, which also indicated that the isolates of *Colletotrichum* spp. have a parasitic rather than mutualistic relationship with the plant. One possible reason is that sterile *A. adenophora* seedlings are inoculated with a high dose (~10^6^ spores/mL) of *Colletotrichum* (see methods), which causes these endophytic *Colletotrichum* fungi in *A. adenophora* to behave physiologically like pathogens. Previous studies have shown that endophytes, like pathogens, can elicit a plant immune response [53], e.g., the induction of the plant hypersensitivity response, including the formation of reactive oxygen species (ROS), the induction of salicylic acid (SA) and jasmonic acid (JA) signaling, and the upregulation of pathogenesis-related (PR) genes and plant defensin 1.2 (PDF1.2) [54,55]. It is valuable to determine whether the expression of immune-related genes such as PR1/2/5 and PDF1.2 is elicited in *A. adenophora* by the colonization of these endophytes in the future.

As in Expectation 2, endophytic *Colletotrichum* fungi had some positive effects on the dominant pathogen Didymellaceae when inoculated on the leaves of *A. adenophora* (Figure 5c,d). Didymellaceae are the dominant foliar pathogens. These pathogens occur widely on *A. adenophora* in the invaded range, and some members exhibit high virulence and a narrow host range [37]. In particular, their virulence evolves with invasion history [56]. Therefore, these pathogens may have important impacts on the expansion of *A. adenophora* along with a delayed invasion history. Our findings indicate that the *Colletotrichum*–Didymellaceae interaction may be important for understanding the role of foliar fungi in plant invasion. Similarly, many studies have verified that foliar fungi can help invasive host plants resist pathogens; for example, foliar endophytic fungi of the invasive plant *Impatiens glandulifera* appear to be antagonistic to rust fungus (*Puccinia komarovii*) [16]. *Cryptosporiopsis quercina* isolated from *Tripterygium wilfordii*, an endophytic fungus, produces a special active peptide, cyclohexenone, which inhibits the growth of the plant pathogens *Sclerotinia sclerotiorum* and *Botrytis cinerea* [57].

Nonetheless, our findings suggest that the mode of action of endophytic fungi against pathogens is much more complex and depends on the different genetic backgrounds of both the endophytic fungi and the specific pathogen being investigated. For example, endophytes of different genotypes presented different resistances to pathogens, with the endophyte AX198 being more active against the pathogens G56 and Y122 than AX39 and AX115 (Figure 5a–c). The resistance of endophytic fungi to different pathogen genotypes also differs, with the endogenous fungi AX39 and AX115 promoting infection by the pathogens *M. ageratinae* G56 and Y122 but weakening infection by the pathogen *M. speciosa* S188 (Figure 5). These results suggested that the resistance caused in the plants of the AX198 strain differed from that of the AX39 and AX115 strains to pathogens, which reflected the differences among strains from different complexes. Second, the results also indicated that AX198 resulted in different LMA responses (Figure 3) and different physiological responses (Figure 1) in *A. adenophora*. Because most *Colletotrichum* strains inhibit the growth of Didymellaceae strains both in vitro and in vivo (unpublished data), the resistance of these endophytic *Colletotrichum* strains may occur through competitive exclusion of the Didymellaceae pathogen. On the other hand, our results also suggest a synergistic interaction between *Colletotrichum* and other pathogens, which results in greater virulence against *A. adenophora*. Similar to our previous study, the foliar fungus *Colletotrichum* sp. promoted the pathogenicity of *Diaporthe helianthi* on *A. adenophora* leaves [30]. Coinfections are common in nature and could increase the pathogenicity of pathogens to the host [58]. For example, compared with inoculation with either of the two pathogens separately, coinfection with *Verticillium dahliae* or *Colletotrichum coccodes* causes more severe foliar disease symptoms and crown rot in potato (Nicola) [59].

The interactions between endophytic microorganisms and plants are influenced by environmental factors, including insufficient nutrient conditions [60] and drought conditions [61]. As in Expectation 3, inoculation with the endophyte *Colletotrichum* alleviated the effects of drought stress and nutrient stress on *A. adenophora* by increasing several growth indicators of *A. adenophora*, including the aboveground biomass, stem length, and chlorophyll content. Environmental stress stimulates the positive effects of endophytes on *A. adenophora* plants and alleviates stress by stimulating plant growth. The response intensity of *A. adenophora* under drought or nutrient stress also varies with the genetic background of these endophytes. The AX198 strain promoted the growth of *A. adenophora* more strongly than the other two strains did (Figure 6 and Figure 7). This result is consistent with strain AX198 being more active against the pathogens G56 and Y122 than AX39 and AX115 are (Figure 5a–c). Moreover, the greater LMA after AX198 inoculation was consistent with these results (Figure 3).

Furthermore, the effects of the endophyte *Colletotrichum* on *A. adenophora* may be dose dependent. The effect is negative under normal hydrothermal conditions but positive under environmental stress conditions. For example, Zhang et al. studied the effects of endophytic fungal infection on the germination and seedling growth of perennial ryegrass under different salt stress levels [62]. They reported that at low concentrations of NaCl, infection with endophytic fungi had little effect on seedling growth, but at high concentrations, the fungus had a significant promoting effect on seedling growth. Cheplick et al. reported that inoculation with endophytic fungi reduced the number of tillers in perennial ryegrass under drought stress, but the number of tillers increased after the recovery period [63]. The endophytic fungus *Colletotrichum tofieldiae* Ct0861, which is a symbiotic fungus of *Arabidopsis thaliana*, promotes plant growth and reproduction only under phosphate starvation conditions [64,65]. In soils with different water-holding capacities, the effects of fungal endophytes on the growth of perennial ryegrass differ because of their different effects under drought conditions [66]. Therefore, it is necessary to explore the interactions between endophytic microorganisms and plants under different strengths of drought and nutrient stresses (including different nutrient element stresses). Moreover, more physiological indicators for stress resistance, including superoxide dismutase (SOD) and catalase (CAT) activity, relative membrane permeability, and malondialdehyde (MDA) content [67,68,69,70,71], are valuable for understanding the detailed physiological changes induced by genetically distinct endophytes in *A. adenophora* under stress conditions. Additional mechanisms by which stress enhances the interaction between fungi and plants are worthy of further exploration in the future.

In general, our findings suggested that three strains of the leaf endophyte *Colletotrichum* did not significantly promote the growth of *A. adenophora* under normal hydrothermal and nutrient conditions; however, these endophytic *Colletotrichum* strains could help *A. adenophora* resist foliar fungal pathogens and improve the response to drought or nutrient stress. Our results provide novel clues to explain why *A. adenophora* contains a high abundance of *Colletotrichum* endophytes [28], but some *Colletotrichum* endophytes have a weak negative impact on the growth of *A. adenophora* [30]. Therefore, *A. adenophora* may be an intermediate host for *Colletotrichum* spp., leading to damage due to invasive plant competition in agricultural crops, as well as damage related to the incidence of diseases caused by *Colletotrichum* spp. Nonetheless, as plant mutualists, microbiomes are not a single organism but a community of species with complex interactions among microbial taxa and between microbes and their shared host. The presence of an individual strain is detrimental to the host, but multiple strains can be beneficial [72]. Therefore, the effects of multiple *Colletotrichum* inoculations on the stress resistance of *Colletotrichum* are also worth studying in the future.

## Data Availability

The original contributions presented in this study are included in the article/Supplementary Material. Further inquiries can be directed to the corresponding author.

## Supplementary Materials

The following supporting information can be downloaded at https://www.mdpi.com/article/10.3390/microorganisms12122565/s1, Figure S1: Planting host *A. adenophora* and inoculation with *Colletotrichum*; Figure S2: Isolation rates of *Colletotrichum* strains from *A. adenophora*; Figure S3: Growth effects of *A. adenophora* inoculated with endophytic *Colletotrichum* strains.
