# Supplementary material for: Role of the Foliar Endophyte Colletotrichum in the Resistance of Invasive Ageratina adenophora to Disease and Abiotic Stress
Source: Microorganisms. 2024 Dec 12;12(12):2565. doi: 10.3390/microorganisms12122565 (PMC11677791; doi:10.3390/microorganisms12122565)
Supplement: Supplementary file 1 [file microorganisms-12-02565-s001.zip › microorganisms-3358956-supplementary.pdf]

Supplementary Material

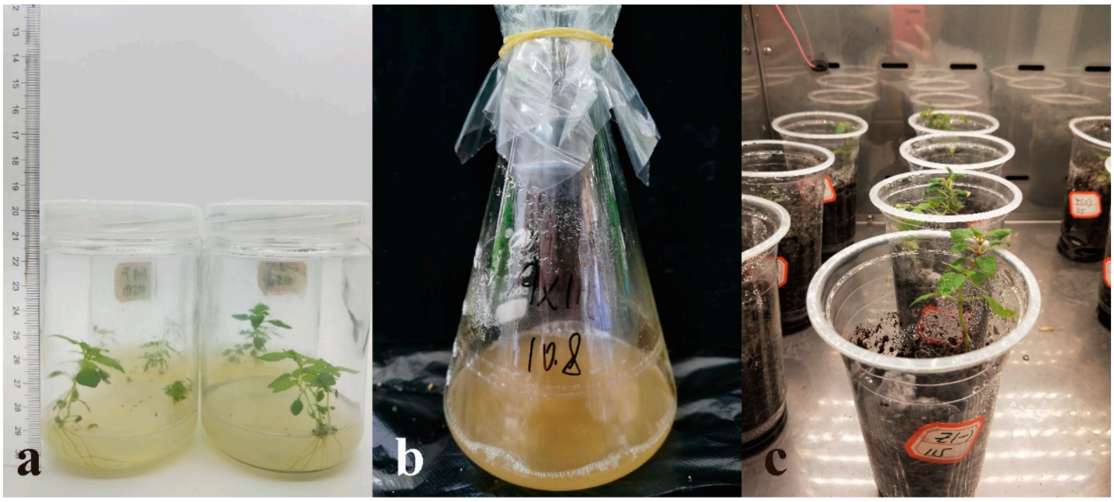

Figure S1 Planting host *A. adenophora* and inoculation with *Colletotrichum*.

a, Sterile seedlings cultured in a tissue culture bottle; b, Preparation of the spore solution of the *Colletotrichum* strain; c, Seedlings transplanted into soils in a plant growth chamber after inoculation with *Colletotrichum*.

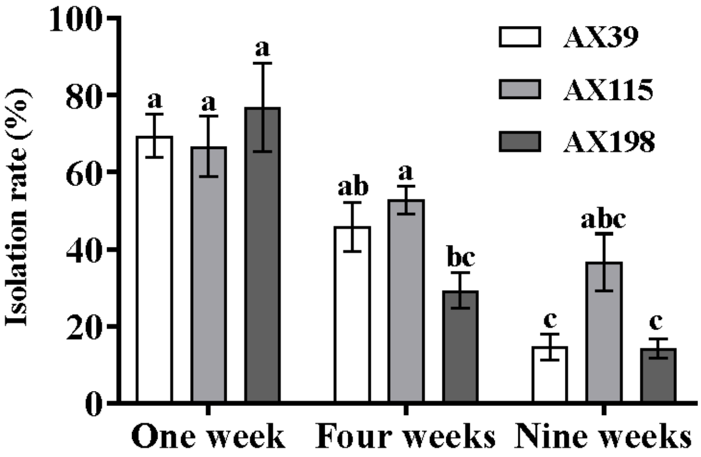

Figure S2 Isolation rates of *Colletotrichum* strains from *A. adenophora*.

Different letters indicate statistically significant (*P<0.05*) differences among different strains at one detection time point. The error bars are the standard errors.

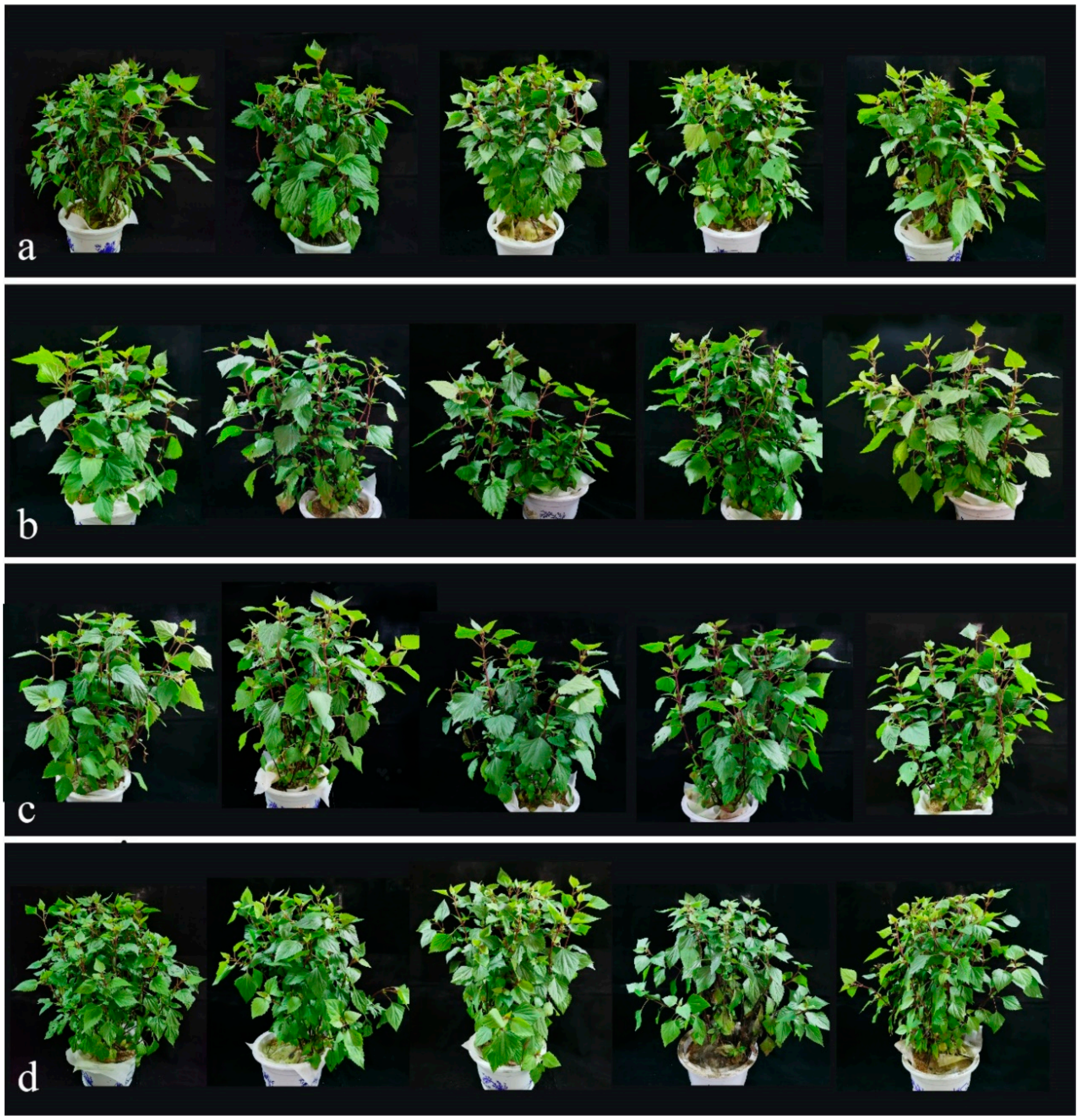

Fig. S3 Growth effects of *A. adenophora* inoculated with endophytic *Colletotrichum* strains. Individuals of *A. adenophora* were inoculated with nothing (a), AX39 (b), AX115 (c), or AX198 (d) and grown for two months in the greenhouse.
